# Associations between types and sources of dietary carbohydrates and liver fat: a UK Biobank study

**DOI:** 10.1186/s12916-023-03135-8

**Published:** 2023-11-16

**Authors:** Josefina Orliacq, Aurora Pérez-Cornago, Siôn A Parry, Rebecca K Kelly, Dimitrios A Koutoukidis, Jennifer L Carter

**Affiliations:** 1 Clinical Trial Service Unit and Epidemiological Studies Unit (CTSU), Nuffield Department of Population Health, University of Oxford, Oxford, UK; 2 Cancer Epidemiology Unit (CEU), Nuffield Department of Population Health, University of Oxford, Oxford, UK; 3 Oxford Centre for Diabetes, Endocrinology and Metabolism, University of Oxford, Oxford, UK; 4 Aston Medical School, Aston University, Birmingham, B4 7ET UK; 5 School of Medicine, College of Health and Medicine, The University of Tasmania, Hobart, Australia; 6 Nuffield Department of Primary Care Health Sciences, University of Oxford, Oxford, UK

**Keywords:** Hepatic steatosis, MASLD, Dietary carbohydrates, Non-alcoholic fatty liver, Carbohydrate quality, Fibre intake

## Abstract

**Background and aims:**

Excess energy intake can lead to metabolic dysfunction-associated steatotic liver disease (MASLD), but the relationship between dietary carbohydrate intake and liver fat content remains unclear. This study aimed to examine the associations between types and sources of dietary carbohydrates and liver fat content.

**Methods:**

UK Biobank participants with no pre-existing diabetes, liver disease or cardiovascular disease reported dietary intake of types and sources of carbohydrates (total carbohydrates, free sugars, non-free sugars, starch from whole grains, starch from refined grains, and fibre) on at least two 24-h dietary assessments. In cross-sectional analyses, (*n* = 22,973), odds ratios (OR) of high liver fat content (defined as a score of ≥ 36 in the hepatic steatosis index) by quintiles of carbohydrate intakes were estimated using multivariable logistic regression models. In prospective analyses, a second sample (*n* = 9268) had liver proton density fat fraction (PDFF) measured by magnetic resonance imaging (2014–2020). Multivariable linear regression models estimated geometric means of PDFF (%) by quintiles of carbohydrate intakes. Models were adjusted for demographic and lifestyle confounders, including total energy intake.

**Results:**

In the cross-sectional analyses, 6894 cases of high liver fat content were identified. Inverse associations between intakes of fibre (OR of highest vs. lowest quintile 0.46 [95% CI: 0.41–0.52]), non-free sugars (0.63 [0.57–0.70]) and starch from whole grains (0.52 [0.47–0.57]) with liver fat were observed. There were positive associations between starch from refined grains and liver fat (1.33 [1.21–1.46]), but no association with free sugars (*p*=0.61). In prospective analyses, inverse associations with PDFF (%) were observed for intakes of fibre (− 0.48 geometric mean difference between highest and lowest quintile of intake [− 0.60 to − 0.35]), non-free sugars (− 0.37 [− 0.49 to − 0.25]) and starch from whole grains (− 0.31 [− 0.42 to − 0.19]). Free sugars, but not starch from refined grains, were positively associated with PDFF (0.17 [0.05 to 0.28]).

**Conclusion:**

This study suggests that different carbohydrate types and sources have varying associations with liver fat, which may be important for MASLD prevention. Non-free sugars, fibre, and starch from whole grains could be protective, while associations with free sugars and starch from refined grains are less clear.

**Supplementary Information:**

The online version contains supplementary material available at 10.1186/s12916-023-03135-8.

## Introduction

Non-alcoholic fatty liver disease (NAFLD) is the most common cause of chronic liver disease in the world [1]. NAFLD, which has been recently redefined as part of steatotic liver disease (SLD) under the term MASLD (metabolic dysfunction-associated fatty liver disease), is a rapidly growing contributor to liver mortality and morbidity globally and affects approximately 25% of the adult population [2–4]. This disease is characterised by the accumulation of fat in the liver [5] and can progress from simple steatosis (≥5.% of liver fat content) to steatohepatitis (≥ 5% of liver fat content and inflammation), and lead to liver fibrosis and cirrhosis [6–8]. These advanced stages are mostly responsible for the substantial economic burden of MASLD [6, 9], and it has been estimated that MASLD will be the first cause of liver transplant by 2030 [3, 10, 11]. To date, the clinical management of MASLD is constrained to lifestyle interventions such as maintaining a healthy weight and balanced diet, as excess energy intake and low energy expenditure are key modifiable risk factors, and at present no pharmacological treatment has been approved [12]. However, it is not yet fully understood how different dietary macronutrients relate to MASLD, independently of energy intake [13, 14].

It has been proposed that dietary carbohydrates increase liver fat accumulation because they promote de novo lipogenesis (DNL), and when this physiological mechanism is stimulated in excess, it would contribute to MASLD [15, 16]. In addition, inflammation has also been pointed as a potential mechanism in which carbohydrates are associated with liver fat accumulation [16]. However, recent studies have suggested that different types and sources of carbohydrates could influence liver fat accumulation differently [13, 17]. Population-based studies looking at total dietary carbohydrate intake and MASLD have observed positive [18–22], negative [23] and non-significant associations [21, 24–29]. A recent meta-analysis of 34 observational studies concluded that there were no significant associations between carbohydrates and MASLD [30]. These inconsistent results may be due to small sample sizes or differences in dietary assessment methods, inclusion criteria, adjustment for confounders or MASLD diagnosis tools. In particular, there are few observational studies that are prospective and have adjusted for total energy intake or assessed different types and sources of carbohydrate intake simultaneously. Therefore, this study sought to study the associations between different types and sources of dietary carbohydrates in the largest prospective study to date with liver fat measured using the most accurate and precise non-invasive method for liver fat quantification, magnetic resonance imaging (MRI) [31].

## Methods

### Study population

The UK Biobank is a large, population-based prospective study of 502,413 participants aged 37–63 years recruited between 2006 and 2010 from 22 assessment centres across Wales, Scotland, and England. During a first assessment visit, participants gave their informed consent and provided detailed information on sociodemographic and lifestyle characteristics and medical conditions via a self-reported questionnaire and an interview [32]. Additionally, anthropometric measures were taken, and blood, urine and saliva samples were collected [33]. A further assessment included multimodal imaging studies, in which a subsample of participants was studied with MRI, from 2016 onwards, which is currently ongoing [34]. The UK Biobank study has complied with all necessary research ethics protocols, according to the Declaration of Helsinki, and approved by the Northwest Multi-Centre Research Ethics Committee (reference number 21/NW/0157) [32]. Further details about this study, including recruitment process and follow-up, can be found online [35].

### Assessment of dietary types and sources of carbohydrate intake

Participants completed up to five 24-h dietary assessments where they indicated their consumption during the last 24 hours from a list of 206 widely consumed foods and 32 beverages [32, 36–38]. The first 24-h assessment was included at the baseline survey at recruitment from 2009 to 2010 (*N* = 70,724). Participants that had provided a valid email address at recruitment were then invited to complete identical 24-h assessments online up to four more times (*N* = 176,012; 53% response rate). See Additional file 1: Fig. S1 for a timing of the assessments and measurements used in this study.

From this questionnaire, carbohydrate intakes from each food item and beverage were automatically calculated by multiplying the frequency of intake by the carbohydrate content of food items and beverages indicated in the UK Nutrient Databank food composition tables [39]. Total carbohydrate intake can be divided into subcategories of sugars, fibre and starch based on the quality of the food sources consumed (see Additional file 1: Fig. S2 for the subdivision of total carbohydrates). ‘Free sugars’ were defined as all added sugars in any form (i.e. sugars added to foods by manufacturer, cook or consumer), plus those naturally present in honey, syrups and unsweetened fruit and vegetable juice [40, 41]. ‘Non-free sugar intake’ refers to naturally occurring sugars found in fruit, vegetables and dairy products; and was calculated by subtracting free sugars from total sugar intake [40]. Separate from the type of sugars contained within the total carbohydrate intake, dietary intake of starch from refined grains and whole grains variables were calculated by combining the grams of starch from specific food products reported. For example, starch from white bread, pizza, and biscuits were included in the ‘starch from refined grains category’, whereas starch from wholemeal bread or oat cereal went into ‘starch from whole grains’ (see Additional file 1: Table S1 for details). Finally, all dietary variables were coded as percentage of total energy intake, except for fibre, which was presented in grams per day, and were categorised into quintiles.

### Assessment of liver fat

Liver fat accumulation was studied both cross-sectionally and prospectively with different outcome assessments.

Cross-sectional associations of dietary carbohydrates and high hepatic steatosis were assessed using a score of ≥ 36 in the hepatic steatosis index (HSI) [42]. This index considers body mass index (BMI), sex, and liver enzymes, and it is calculated as: (ALT/ AST) *8 + BMI + 2 if female, and + 2 if diabetic. The HSI at a cut-off value of 36 has shown varying sensitivity (46–89.5%) and higher specificity (60–95.2%) in identifying steatosis cases, when validated in populations with different underlying conditions [42–45].

ALT and AST enzymes were obtained from the baseline visit blood samples [46]. At least one of the 24-h dietary questionnaires had to be taken at baseline, when blood samples and BMI measurements were taken.

The prospective analyses were carried out in a second sample that measured liver fat by MRI, which provides an estimate of the liver proton density fat fraction (PDFF) in terms of liver fat percentage (%). The MRIs were measured on average 6 years and 3 months after the final 24-h dietary questionnaire was collected (Additional file 1: Fig. S1).

### Inclusion and exclusion criteria

Participants with cardiovascular disease at baseline were excluded due to its association with MASLD and to reduce reverse causality because medical advice likely includes changes to diet. Participants with conditions that alter liver enzymes or liver metabolism significantly were also excluded, such as chronic liver disease, diabetes, dyslipidaemia, severe endocrine conditions, pregnancy, or use of medication that can promote liver inflammation [47] (Additional file 1: Table S2 and Fig. S3) Participants in the highest category of alcohol consumption according to NICE guidelines (higher risk drinkers, i.e. > 50 alcohol units per week in men and > 30 in women) were excluded to prevent associations of liver fat with high alcohol consumption [48]. Finally, participants were excluded that had missing data on liver fat, reported implausible energy intake (total energy intake of < 600 or > 3500 kcal/day in women, and < 800 or < 4200 kcal/day in men) or less than two 24-h dietary assessments [49]. The final cross-sectional sample was *N* = 22,973 participants, and the prospective analyses had* N* = 9268 participants remaining with MRI measurements.

### Statistical analysis

In cross-sectional analyses, multivariable logistic regression models were built to estimate the odds of high liver fat across quintiles of carbohydrate types and sources. Potential confounders were added sequentially, and model fit improvement was checked with likelihood ratio tests between nested models with and without additional covariates.

Covariates were included in the following order: sex, age, ethnicity (5 categories), Townsend deprivation index [49] (quintiles), education (university or equivalent, A-levels, GCSE, none/unknown), region (10 categories), smoking status (never, former, current), physical activity (low, [< 10 metabolic equivalent of task (MET) h/week], moderate [10–50 MET h/week], high [>50 MET h/week]) [50], total energy intake (quintiles, kilojoules [kJ]) and polyunsaturated fatty acids (PUFA)/saturated fatty acids (SFA) ratio, as a marker of a healthy diet [51] (see Additional file 1: Table. S3 for details). BMI was not adjusted for in cross-sectional analyses as it was included as part of the HSI.

For the prospective analyses, multivariable linear regression models estimated geometric means of PDFF by quintiles of carbohydrate types and sources intake. PDFF had a skewed distribution and was transformed by using its natural logarithm (ln), and robust standard errors were calculated. Adjusted means of PDFF were predicted from the resulting beta coefficients and were exponentiated to calculate the geometric mean for each quintile. To help interpret a change in geometric means, the geometric mean change in PDFF was also expressed as a percentage change (Additional file 1: Table S4 ). Confounders in the prospective models were added in the same order as in the cross-sectional analyses, although there was a further adjustment for BMI (categorised following the international classification for adults aged over 20 years [51], which were defined as < 25, 25–29.9, 30–34.9 and ≥ 35). To assess if the associations between dietary exposures and liver fat were significantly different across BMI groups and sex, an interaction term was included in the regressions, and a likelihood ratio test was used to assess model improvement. There was no violation of the assumptions of the linear regression model.

To assess the robustness of the results, a sensitivity analysis including only participants who completed at least four 24-h recall dietary questionnaires was conducted. This was done to observe the impact of measurement error due to potential exposure misclassification. Additional sensitivity analyses assessed the impact of adjusting for diagnosed hypertension, and the impact on cross-sectional analyses of using a larger sample of anyone who completed at least two 24-h dietary assessments (rather than restricting to participants who completed their first 24-h dietary assessment at the same time that liver enzymes were measured).

All analyses were done using STATA S.E 16 (StataCorp. 2019. Stata Statistical Software: Release 16. College Station, TX: StataCorp LLC), and the significance level was considered *p* < 0.05. Forest plots were produced using R package ‘Jasper makes plots’ [52].

## Results

### Cross-sectional analyses between carbohydrate intake and odds of high liver fat

In the cross-sectional analysis, 6894 participants were classified as presenting high liver fat content by HSI (32%), (Table 1). Participants with high liver fat content were more likely to be current smokers, and in the low physical activity category compared to those with low-to-moderate liver fat. Cases of high liver fat also had a higher consumption of starch from refined grain, and a lower consumption of fibre, starch from wholegrains and non-free sugars compared to controls.

**Table 1:** Characteristics of participants in the cross-sectional analyses, by cases of high HSI (*N* = 22,973)

**Characteristics**	**Controls (*N* = 16,079)**	**High HSI**^**a**^** (*N* = 6894)**	**Total (*N* = 22,973)**	***p* value**^**b**^
**Age (years)**^**c**^	55.1 (8.1)	54.8 (7.8)	55.0 (8.0)	0.004^d^
**Male sex (%)**	6021 (37)	2769 (40)	8790 (38)	< 0.001
**Body mass index (kg/m**^**2**^**)**^**c**^	24.1 (2.5)	31.0 (4.1)	26.2 (4.4)	< 0.001
**White ethnicity (%)**	15,406 (95.8)	6536 (94.8)	21,942 (95.5)	0.001
**Most deprived quintile (%)**	3109 (19.4)	1480 (21.5)	4589 (20.0)	< 0.001
**University degree (%)**	12,224 (76.0)	4845 (70.3)	17,069 (74.3)	< 0.001
**Current smoker (%)**	855 (5.3%)	436 (6.3)	1291 (5.6)	< 0.001
**Low physical activity (%)**	2553 (15.9)	1775 (25.7)	4328 (18.8)	< 0.001
**Hazardous alcohol intake (%)**	6,151 (38.3)	2575 (37.4)	8726 (38.0)	< 0.001
**Alcohol intake (units/week)**^**e**^	12 (4, 20)	11.5 (2.8, 22)	12 (4, 20.5)	0.003^f^
**ALT/AST ratio**^**e**^	0.72 (0.61, 0.85)	1.01 (0.84, 1.22)	0.79 ( 0.65, 0.97)	< 0.001^f^
**PUFA/SFA ratio**^**e**^	0.49 (0.39, 0.62)	0.48 (0.39, 0.61)	0.49 (0.39, 0.61)	< 0.001^f^
**Total energy intake (kJ)**^**c**^	8321.8 (1870.7)	8350.8 (1956.1)	8330.5 (1896.8)	0.29^d^
**Total carbohydrates (% energy)**^**c**^	51.4 (7.0)	50.6 (7.2)	51.1 (7.1)	< 0.001^d^
**Free sugars (% energy)**^**c**^	11.6 (4.8)	11.8 (5.1)	11.7 (4.9)	< 0.001^d^
**Non-free sugars (% energy)**^**c**^	14.0 (5.6)	13.1 (5.6)	13.7 (5.6)	< 0.001^d^
**Starch from whole grains (% energy)**^**c**^	11.8 (6.1)	12.7 (6.4)	12.0 (6.2)	< 0.001^d^
**Starch from refined grains (% energy)**^**c**^	5.9 (4.3)	4.8 (3.9)	5.6 (4.2)	< 0.001^d^
**Fibre (g/day)**^**c**^	18.5 (5.7)	17.2 (5.5)	18.1 (5.7)	< 0.001^d^

Values are presented as *N* (proportion)

^a^Defined by a score of 36 in the Hepatic steatosis index

^b^*P* values represent chi-squared test

^c^*Values are mean (standard deviation)*

^d^*P values represent analysis of variance*

^e^Values are median (interquartile range)

^f^ P values represent Wilcoxon rank-sum test

**Fig. 1:**
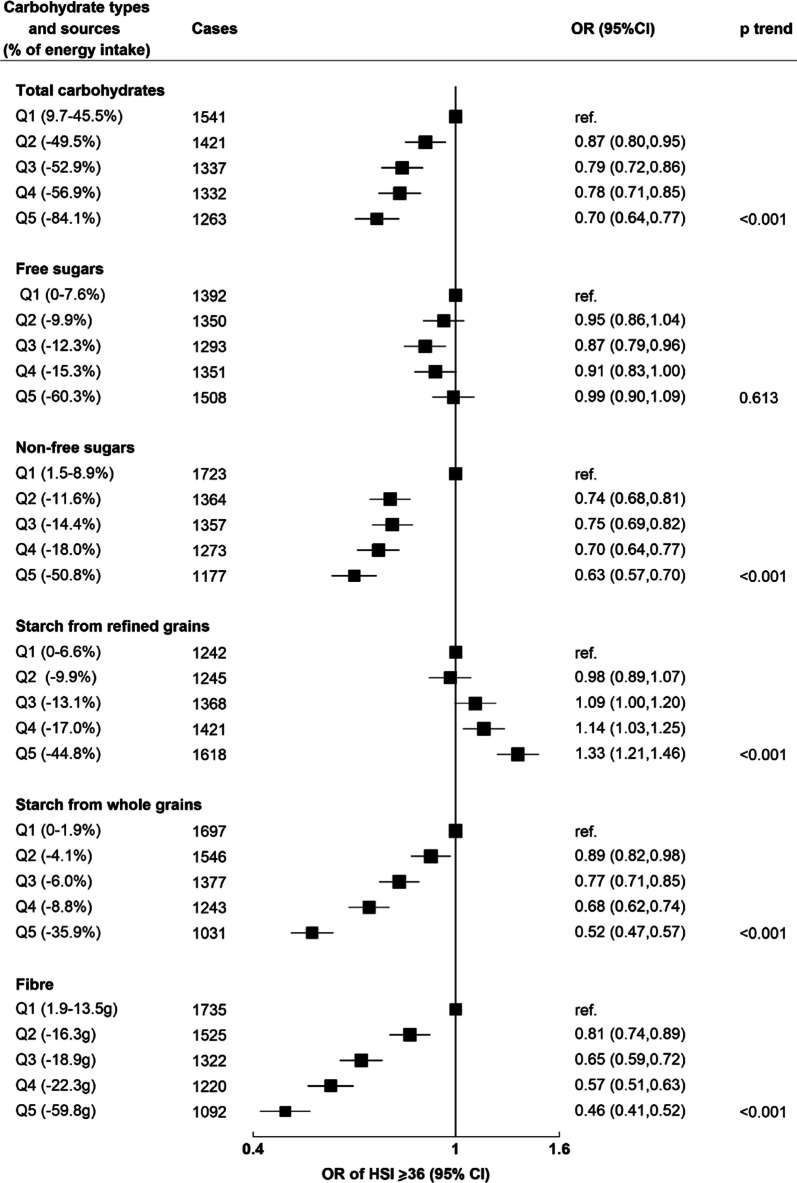
Cross-sectional results (*N* = 22,973). Odds ratio of steatosis, defined as ≥36 HSI score. Results from logistic regression models, adjusted by age, sex, ethnicity, deprivation, education, smoking status, physical activity, alcohol intake, total energy intake and PUFA:SFA ratio

In the fully adjusted cross-sectional analyses, the highest quintile of total carbohydrate intake was associated with 30% lower odds of high liver fat compared to the lowest quintile (OR: 0.70, 95% confidence interval 0.64–0.77, *p* for trend < 0.001; Fig. 1). Free sugars were not significantly associated with high liver fat content (*p* for trend = 0.61), although non-free sugars were associated with 37% lower odds of high liver fat for participants in the highest vs. lowest quintile of intake (0.63 [0.57–0.70]; *p* for trend < 0.001). The highest quintile of starch from refined grains had a positive association with high liver fat content compared to the lowest quintile (OR: 1.33 [ 1.21–1.46]), whereas both starch from whole grains and fibre reported strong inverse linear associations with high liver fat, with 48% and 54% lower odds reported for quintile 5 compared to quintile 1, respectively (0.52 [0.47–0.57]; 0.46 [0.41–0.52]).

### Prospective analyses between carbohydrate intake and average liver fat percentage

In the prospective sample, the PDFF geometric mean on the first imaging assessment was 2.6% (1.9–4.0) and 1416 (15%) participants had steatosis (>= 5.6% PDFF) [8]. Participants in the highest quintiles of non-free sugars, starch from wholegrains and fibre tended to be university-educated and less likely to smoke or engage in low physical activity, but the differences were small compared to the first quintile (Table 2).

**Table 2:** Participant characteristics in the prospective analyses, across lowest and highest quintiles of intake of carbohydrate types and sources (*N* = 9268)

		**Total carbohydrates**		**Free sugars**		**Non-free sugars**		**Starch from refined grains**		**Starch from whole grains**		**Fibre**		**Overall**
		**Q1**	**Q5**	**Q1**	**Q5**	**Q1**	**Q5**	**Q1**	**Q5**	**Q1**	**Q5**	**Q1**	**Q5**	
**Liver fat %**^a^		2.7 (1.9, 4.4)	2.5 (1.8, 3.8)	2.5 (1.8,3.8)	2.7 (1.9,4.4)	2.9 (2.0,5.0)	2.4 (1.8,3.5)	2.5 (1.8,3.8)	2.6 (1.9,4.2)	2.8 (1.9,4.6)	2.4 (1.8,3.6)	2.8 (1.9,4.7)	2.5 (1.8,3.7)	2.6 (1.9,4.0)
**Age, mean (SD)**		53 (7)	53 (7)	53 (7)	52 (7)	51 (7)	55 (7)	55 (7)	51 (7)	52 (7)	54 (7)	52 (7)	54 (7)	53 (7)
**Male sex**		784 (42)	660 (35)	550 (29)	908 (49)	1,072 (57)	454 (24)	337 (43)	411 (52)	745 (40)	796 (43)	689 (37)	878 (47)	3738 (40)
**BMI**	**<25**	776 (42)	938 (51)	854 (46)	858 (46)	758 (41)	963 (52)	917 (50)	866 (47)	802 (43)	1008 (54)	770 (42)	944 (51)	4439 (48)
	**25–**	805 (43)	707 (38)	706 (38)	768 (41)	822 (44)	644 (35)	718 (39)	728 (39)	749 (40)	657 (35)	793 (43)	692 (37)	3642 (39)
	**30–**	219 (12)	162 (9)	239 (13)	185 (10)	222 (12)	193 (10)	179 (10)	211 (11)	234 (13)	150 (8)	231 (13)	174 (9)	961 (10)
	**35–**	53 (3)	43 (2)	53 (3)	40 (2)	52 (3)	53 (3)	40 (2)	45 (2)	68 (4)	37 (2)	59 (3)	41 (2)	221 (2)
**White ethnicity**		1813 (98)	1770 (96)	1781 (96)	1772 (96)	1796 (97)	1782 (96)	1805 (97)	1752 (95)	1774 (96)	1797 (97)	1776 (96)	1804 (97)	8985 (97)
**Most deprived quintile**		393 (21)	388 (21)	394 (21)	411 (22)	427 (23)	349 (19)	331 (18)	432 (23)	420 (23)	389 (21)	397 (21)	364 (20)	1849 (20)
**University degree**		1517 (82)	1486 (80)	1490 (80)	1451 (78)	1442 (78)	1494 (81)	1505 (81)	1461 (79)	1461 (79)	1497 (81)	1387 (75)	1544 (83)	7,456 (80)
**PUFA/SFA ratio, mean (SD)**		0.5 (0.2)	0.6 (0.2)	0.6 (0.2)	0.5 (0.2)	0.5 (0.2)	0.6 (0.2)	0.6 (0.2)	0.5 (0.2)	0.5 (0.2)	0.6 (0.2)	0.5 (0.2)	0.6 (0.2)	0.5 (0.2)
**Current smoker**		117 (6)	76 (4)	84 (4)	114 (6)	140 (8)	61 (3)	83 (4)	99 (5)	143 (8)	61 (3)	130 (7)	74 (4)	436 (5)
**Low physical activity**		392 (21)	331 (18)	367 (20)	386 (21)	438 (24)	304 (16)	341 (18)	429 (23)	411 (22)	330 (18)	477 (26)	266 (14)	1,837 (20)
**Hazardous alcohol intake**		1,145 (62)	403 (22)	743 (40)	671 (36)	987 (53)	508 (27)	789 (43)	687 (37)	809 (44)	649 (35)	795 (43)	685 (37)	3,780 (41)
**Alcohol intake (units/week)**^a^		20 (12, 28.5)	10 (4, 16)	14 (8, 24)	12.5 (6, 23)	18 (10, 28)	11 (4.5, 18)	14.5 (8, 24)	12.5 (6, 21.5)	15 (8, 24.5)	12.5 (6.5, 20.5)	14.5 (8, 24)	13.5 (6.5, 23)	14 (8, 23)
**Total energy intake (kJ)**, **mean(SD)**		8529 (1986)	7861 (1797)	7789 (1740)	8685 (1988)	9040 (1999)	7606 (1710)	7992 (1847)	8473 (1905)	8361 (1998)	8072 (1787)	7005 (1563)	9792 (1918)	8400 (1894)

Summary statistics are presented as *N* (%) for categorical variables, which include a missing value variable, unless otherwise stated

^a^Values are median (IQR). BMI: body mass index. Hazardous alcohol intakes are defined as >14 units per week, < 35 in women and <50 in men. Low physical activity is defined as < 10 metabolic equivalent of task hours per week. *kJ* kilojoules

**Fig. 2:**
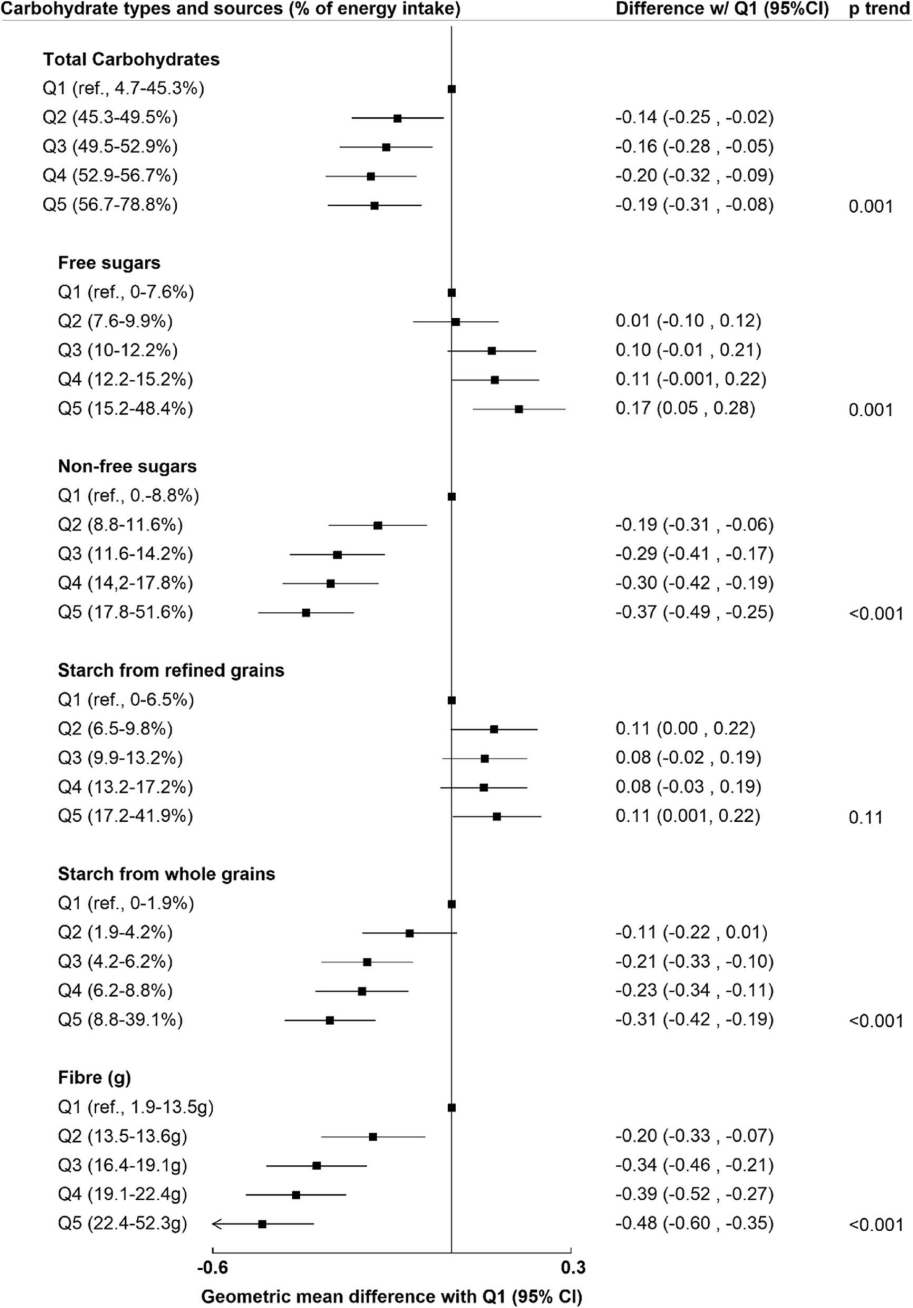
Prospective analyses (*n* = 9268). Geometric mean difference in liver fat between the highest (Q5) and lowest quintile (Q1) of dietary intake of carbohydrate types and sources. Results from fully adjusted linear regression models, controlling for age, sex, ethnicity, deprivation, education, region, smoking status, physical activity, alcohol intake, PUFA/SFA ratio, and body mass index

In the fully adjusted prospective analyses (Fig. 2), after adjusting for BMI, total carbohydrates had an inverse association with PDFF, but the association was not linear; all groups reported a similar decrease in the average PDFF in comparison with quintile 1. Free sugar intake was positively associated with PDFF: there was a 0.17 mean difference in PDFF between the highest and lowest quintiles of intake (95% CI, 0.05–0.28). Meanwhile, non-free sugars were inversely associated with average PDFF to a greater extent (− 0.37 comparing quintile 5 to quintile 1; − 0.49 to − 0.25). Starch from refined grains was not associated with PDFF (*p* for trend = 0.11), but there were linear inverse associations for both starch from wholegrains and fibre with PDFF (*p* for trend < 0.001 for both). The absolute mean difference between quintiles 5 and 1 of starch from wholegrains was − 0.31 (− 0.42 to − 0.19), which was weaker than the absolute difference between extreme quintiles for fibre (− 0.48 [− 0.60 to − 0.35]). If the difference in geometric means was translated to a percentage change in PDFF, the highest quintile of fibre intake was associated with approximately a 17% lower PDFF compared to quintile 1 (Additional file 1: Table S4).

The sequential adjustment for confounders and their impact in the associations can be seen in Additional file 1: Tables S5 and S6.

There was no evidence of heterogeneity by sex (Additional file 1: Table S7). but there was evidence of interaction between BMI groups and starch from refined grains and non-free sugars (*p*_heterogeneity_ = 0.01 and *p*_heterogeneity_ = 0.001, respectively), where the associations were stronger at greater levels of BMI (Additional file 1: Table S8). In the sensitivity analyses with only those that had answered at least four 24-h dietary assessments, the cross-sectional sample was reduced to *N* = 9046 participants and 2488 cases (Additional file 1: Tables S9 and S10) and the prospective analysis was reduced to 2829 participants and 404 cases (14.3%). Generally, similar patterns were seen across types and sources of carbohydrates for both cross-sectional and prospective analyses, with slightly stronger results for non-free sugars, starch from wholegrains and fibre in the sensitivity analyses than in the main analyses (e.g. fibre quintile 5 vs quintile 1 OR: 0.35 in the sensitivity analysis, vs 0.46 in the main analysis). Sensitivity analyses adjusting for diagnosed hypertension did not affect the cross-sectional associations although the inverse associations in prospective analyses became slightly stronger (Additional file 1: Tables S11 and S12). Conducting cross-sectional analyses on a larger sample of any participants that had at least two 24-h dietary assessments (rather than restricting the sample to at least one 24-h dietary assessment when liver enzymes were measured) did not materially affect most associations with carbohydrate intake, although free sugars became weakly inverse (Additional file 1: Table S13).

## Discussion

In the largest observational investigation of macronutrient intake and liver fat to date, associations varied across different types and sources of carbohydrates with liver fat accumulation. Overall, the results from both the cross-sectional and prospective analyses suggested strong inverse and independent associations between the intake of non-free sugars, fibre and starch from whole grains with liver fat. Conversely, free sugars were positively associated with liver fat in the prospective analyses but not the cross-sectional analyses, whereas starch from refined grains was not associated with liver fat in prospective analyses but displayed positive associations in the cross-sectional analyses.

Total carbohydrates were inversely associated with liver fat in both cross-sectional and prospective analyses, albeit weakly in the latter. However, other observational studies from Japan, Iran and Korea reported positive associations between high carbohydrate intake and measurements of liver fat, possibly due to differences in the proportions of subtypes of carbohydrates typically consumed in different populations compared to the UK [18–21]. Since other studies from European populations have likewise found inverse associations with carbohydrate intake akin to the current study, it overall suggests that mixed results may be due to variations in the types and sources of carbohydrate, which were not measured in these previous studies [23].

When looking in more detail at the types and sources of carbohydrates, the current study found strong, inverse associations between fibre and starch from wholegrains with high liver fat in both the cross-sectional and prospective analyses. Cross-sectional and case-control studies in America and Europe also previously reported inverse associations with fibre, although the current study is the first to show large-scale prospective evidence with MRI-based phenotyping of liver fat [23, 24]. Fibre, which wholegrains contain high amounts of, may reduce low-grade inflammation, improve lipid profiles, increase satiety and supress ghrelin, a hormone with orexigenic effects; this could explain why it is negatively associated with liver fat [14, 53]. In addition, it may affect the gut microbiome, by influencing the gut barrier, gastrointestinal immune and endocrine responses, thereby playing a role in whole-body and liver metabolism [54].

In contrast, the associations with starch from refined grains were less clear, with a weakly positive relationship suggested from the cross-sectional analyses but a generally flat association in the prospective results. A previous systematic review and meta-analysis of observational studies concluded there was not a significant relationship between refined grains and MASLD, although none of these studies were prospective [55]. Meanwhile, a recent RCT of 50 overweight adults with a 12-week feeding intervention of refined grains reported a 49% increase in liver fat [56]. While the RCT was small and susceptible to chance findings, there is mechanistic evidence suggesting that refined starchy foods may cause the accumulation of fat in the liver by promoting inflammation [16]. Our population-based prospective study suggests that the association of liver fat with starch may vary by the source of starch, although while the benefit of fibre was able to be detected, more research is needed to verify whether consuming more refined grains is harmful. It could be that the healthy volunteer bias of UK Biobank may have weakened any risks associated with the consumption of refined grains, and more large-scale prospective studies will need to assess different sources of starch and liver fat accumulation [50]. Alternatively, the prospective analyses included an adjustment for BMI that was not done in cross-sectional analyses as this variable was contained in the HSI index; this may have contributed to the differing results across time points, if BMI is the main pathway through which starch from refined grains or free sugars is associated with liver fat.

The inverse linear association demonstrated here with non-free sugars is novel, and to the best of our knowledge, no previous study has looked at the relationship between this exposure and liver fat. Sources of non-free sugars may be high in fibre, such as vegetables and fruits - but non-free sugars also come from dairy, which is low in fibre content. This suggests that their role in liver fat accumulation could be independent from fibre. Recent research has also shown an inverse association between dairy products and type 2 diabetes, another important metabolic condition that is also associated with MASLD [57, 58]. Further research could focus on sources of non-free sugars to understand whether they have different associations with liver fat.

On the other hand, the associations between free sugars and high liver fat were non-significant in the cross-sectional analyses, but positive in the prospective analyses. While the cross-sectional analyses had more power due to sample size, the prospective analysis had a more reliable outcome ascertainment. Previous research is likewise mixed, with a review of observational studies suggesting a positive association, whereas both positive and null results have been reported from RCTs of dietary intervention trials [59–61]. Some of the differences may be due to variation in food groups comprising the term ‘free sugars’, with research on free sugars from sugar-sweetened beverages (SSBs) generally more consistently associated with an increase in liver fat than free sugars from other sources [59–61]. A recent meta-analysis of controlled trials concluded that excess energy from SSBs is associated with large increases in liver fat [62]. Therefore, looking at the association of free sugar intake with liver fat as part of an isocaloric or hypercaloric diet is also an important source of variation in the previous research, and more research needs to assess if sources of free sugars besides those from beverages are associated with liver fat accumulation, independent of overall energy consumption.

This study had several strengths, such as studying the exposure of dietary carbohydrates as a whole group and as types and sources simultaneously. This was possible due to the availability of detailed dietary data from the Oxford WebQ. Previous research has shown that the dietary assessment methods in the UK Biobank estimate intake with acceptable reproducibility and validity, with the advantage of being feasible to administer in a large population without too much participant burden [38, 63]. The exclusion of participants with underlying health conditions helped attenuate the influence of reverse causality, although we cannot fully rule out reverse causality whereby subclinical disease may have led the participants to change their diet prior to measurement in this study. Many potential confounders were also adjusted for in the analysis, including energy intake, although the calculation of an E-value indicated that unmeasured confounders with associations of 2.31 with both the exposure and outcome could explain away the inverse relationship of fibre with the odds of liver fat in the cross-sectional analyses [64, 65].

A key limitation in this study is the potential selection bias arising from the low response rate in UK Biobank (5%), which may have introduced a healthy volunteer bias particularly for those who agreed to answer two or more dietary questionnaires (or four, as in sensitivity analyses) [32, 63]. However, research has shown that even with such a low response rate, estimated risk factor associations with disease in the UK Biobank appeared reliable [66–69]. Carbohydrate intakes were calculated from self-reported questionnaires, and key confounders like physical activity were estimated from self-reported questionnaires which may have introduced measurement error and information bias [38]. For example, the 24-h dietary assessment used here does not collect information about food items that were not on the list, which could lead to an underestimation of dietary intake and lead to residual confounding. Using at least two 24-h dietary assessments and removing implausible intakes attempted to minimise this information bias. Importantly, some subtypes of carbohydrates have more within-person variability than others: previous research indicated that within-person variability may be larger for starch than for fibre in UK Biobank 24-h assessments [70]. Within-person variability in exposures will introduce random error that leads to regression dilution bias and attenuates associations with disease towards the null, and this bias will be greater in the types of carbohydrates that had more variability [71]. Lastly, the outcome of HSI in cross-sectional analyses was an indirect proxy of liver fat that has not been validated in a UK population and is driven mostly by BMI, which was already high in this population. Thus, while an overestimation of hepatic steatosis using the HSI in this population may be a limitation of the cross-sectional analysis, using an index for hepatic steatosis in the baseline sample of UK Biobank allowed for the large-scale investigation of carbohydrate quality and MASLD, with measurements on approximately 23,000 participants.

It is important to note that in this paper we use the term MASLD when referring to previous data that originally used the term NAFLD. This was done in order to adopt the new nomenclature that has been introduced this year [4]. While the new definition is slightly different, and includes the presence of one metabolic factor, a recent study showed that it is possible to consider NAFLD cases data as MASLD cases [72].

## Conclusions

This study suggests that variations in carbohydrate types and sources may contribute differently to liver fat: non-free sugars, fibre, and starch from whole grains could be protective of liver fat accumulation, while associations with free sugars and starch from refined grains are less clear and may be harmful. While this study cannot establish causality, it can guide future research to understand how carbohydrates may have diverse roles in the risk of MASLD. Due to the public health relevance of this disease, and the need for strong evidence to guide dietary advice to prevent it, further research is needed that focuses on carbohydrate types and sources to prevent MASLD at stages in which it is still reversible.

### Supplementary Information

**Additional file 1.** **Table S1.** Data-field codes used to estimate dietary variables. **Table S2.** Exclusion criteria. **Table S3.** Data-fields used for variables included in the analyses. **Table S4.** Difference with liver fat geometric mean in quintile 1, presented as percentage (%). Prospective analyses (*N*=9,268). **Table S5.** Cross-sectional analyses (*N*=22,973) ORs of HSI≥36, by quintiles of intake of carbohydrate types and sources with sequential adjustment for confounders. **Table S6.** Sequential adjustment for confounders (*N*=9,268). **Table S7.** Test for heterogeneity by sex. **Table S8.** Test for heterogeneity across groups of Body Mass Index (BMI). Estimated change in mean liver fat percentage per 1% increase of nutrient, by groups of BMI, obtained from fully adjusted linear regression models. **Table S9.** Sensitivity analyses of participants who answered at least four 24-hr dietary assessments in cross-sectional analyses (*N*=9,046). ORs of HSI≥36, by quintiles of carbohydrates types and sources. **Table S10.** Sensitivity analyses of participants who answered at least four 24-hr dietary assessments in prospective analyses (*N*=2,829). **Table S11.** Cross sectional analyses (*N*=22,793), with additional adjustment for diagnosed high blood pressure^^^. Odds ratio of HSI>36 (95%CI). **Table S12.** Prospective analyses (*N*=9,268), with additional adjustment for diagnosed high blood pressure. **Table S13.** Analyses restricted to participants who answered a minimum of 2 WebQs, taken at any point. (*N*=81,801). **Table S14.** Associations between individual components of the Hepatic Steatosis Index and types and sources of dietary carbohydrates, by quintiles of intake. **Figure S1.** Timeline of UK Biobank data collection relevant to this study’s exposures and outcomes. **Figure S2.** Types and sources of dietary carbohydrates used as exposures for this study. **Figure S3.** Flowchart of UK Biobank showing exclusion of participants included in the cross-sectional and prospective samples.

## Data Availability

All results from this analysis are returned to the UK Biobank within 6 months of publication, at which point they can be made available to other researchers upon reasonable request. UK Biobank is an open-access resource, and researchers can apply to use the dataset at http://ukbiobank.ac.uk/register-apply/. The study protocols are published online at https://www.ukbiobank.ac.uk/learn-more-about-uk-biobank/about-us and https://doi.org/10.1093/ije/dyu089. This study was conducted under CEU-wide UK Biobank unit Application Reference Number 67506. The statistical analysis plan and analytic code are available upon request to the corresponding author.

## Abbreviations

ALT: Alanine aminotransferase
AST: Aspartate aminotransferase
HSI: Hepatic steatosis index
MASLD: Metabolic dysfunction-associated steatotic liver disease
NAFLD: Non-alcoholic fatty liver disease
PDFF: Liver proton density fat fraction (%)
