# Supplementary material for: Associations between types and sources of dietary carbohydrates and liver fat: a UK Biobank study
Source: BMC Med. 2023 Nov 16;21:444. doi: 10.1186/s12916-023-03135-8 (PMC10652437; doi:10.1186/s12916-023-03135-8)
Supplement: Supplementary file 1 — Additional file 1. Table S1. Data-field codes used to estimate dietary variables. Table S2. Exclusion criteria. Table S3. Data-fields used for variables included in the analyses. Table S4. Difference with liver fat geometric mean in quintile 1, presented as percentage (%). Prospective analyses (N=9,268). Table S5. Cross-sectional analyses (N=22,973) ORs of HSI≥36, by quintiles of intake of carbohydrate types and sources with sequential adjustment for confounders. Table S6. Sequential adjustment for confounders (N=9,268). Table S7. Test for heterogeneity by sex. Table S8. Test for heterogeneity across groups of Body Mass Index (BMI). Estimated change in mean liver fat percentage per 1% increase of nutrient, by groups of BMI, obtained from fully adjusted linear regression models. Table S9. Sensitivity analyses of participants who answered at least four 24-hr dietary assessments in cross-sectional analyses (N=9,046). ORs of HSI≥36, by quintiles of carbohydrates types and sources. Table S10. Sensitivity analyses of participants who answered at least four 24-hr dietary assessments in prospective analyses (N=2,829). Table S11. Cross sectional analyses (N=22,793), with additional adjustment for diagnosed high blood pressure^. Odds ratio of HSI>36 (95%CI). Table S12. Prospective analyses (N=9,268), with additional adjustment for diagnosed high blood pressure. Table S13. Analyses restricted to participants who answered a minimum of 2 WebQs, taken at any point. (N=81,801). Table S14. Associations between individual components of the Hepatic Steatosis Index and types and sources of dietary carbohydrates, by quintiles of intake. Figure S1. Timeline of UK Biobank data collection relevant to this study’s exposures and outcomes. Figure S2. Types and sources of dietary carbohydrates used as exposures for this study. Figure S3. Flowchart of UK Biobank showing exclusion of participants included in the cross-sectional and prospective samples. [file 12916_2023_3135_MOESM1_ESM.docx]

**Additional file 1**

**Associations between types and sources of dietary carbohydrates and liver fat: a UK Biobank study**

Josefina Orliacq^1^, Aurora Pérez-Cornago^2^, Siôn Parry^3^, Rebecca Kelly^2^, Dimitrios Koutoukidis^4^, Jennifer L Carter^1^ *

^1^ Clinical Trial Service Unit and Epidemiological Studies Unit (CTSU), Nuffield Department of Population Health, University of Oxford, Oxford, UK

^2^ Cancer Epidemiology Unit (CEU), Nuffield Department of Population Health, University of Oxford, Oxford, UK

^3^Oxford Centre for Diabetes, Endocrinology and Metabolism, University of Oxford; Oxford, UK.

^4^ Aston Medical School, Aston University, Birmingham, B4 7ET, UK.

^5^ School of Medicine, College of Health and Medicine, The University of Tasmania, Hobart, Australia

^6^ Nuffield Department of Primary Care Health Sciences, University of Oxford, Oxford, UK

**Additional file 1**

Tables

**[Table S1.](#_Toc148100239)** [Data-field codes used to estimate dietary variables 3](#_Toc148100239)

[**Table S2.** Exclusion criteria 4](#_Toc148100240)

[**Table S3.** Data-fields used for variables included in the analyses 5](#_Toc148100241)

[**Table S4.** Difference with liver fat geometric mean in quintile 1, presented as percentage (%). Prospective analyses (N=9,268) 6](#_Toc148100242)

[**Table S5**. Cross-sectional analyses (N=22,973) of the odds ratio (OR) and 95% confidence intervals (95%CI) of HSI≥36, by quintiles of intake of carbohydrate types and sources with sequential adjustment for confounders. 7](#_Toc148100243)

[**Table S6.** Sequential adjustment for confounders (N=9,268). Difference with geometric mean of liver fat in Q1 of types and sources of carbohydrates, obtained from linear regression models. 8](#_Toc148100244)

[**Table S7.** Test for heterogeneity by sex 9](#_Toc148100245)

[**Table S8**. Test for heterogeneity across groups of Body Mass Index (BMI). Estimated change in mean liver fat percentage per 1% increase of nutrient, by groups of BMI, obtained from fully adjusted linear regression models. 9](#_Toc148100246)

[**Table S9**. Sensitivity analyses of participants who answered at least four 24-hr dietary assessments in cross-sectional analyses (N=9,046). ORs of HSI≥36, by quintiles of carbohydrates types and sources.. 10](#_Toc148100247)

[**Table S10.** Sensitivity analyses of participants who answered at least four 24-hr dietary assessments in prospective analyses (N=2,829). 10](#_Toc148100248)

[**Table S11.** Cross sectional analyses (N=22,793), with additional adjustment for diagnosed high blood pressure. Odds ratio of HSI>36 (95%CI) 11](#_Toc148100249)

[**Table S12.** Prospective analyses (N=9,268), with additional adjustment for diagnosed high blood pressure^^^. Difference with liver fat geometric mean in quintile 1 (95%CI) 11](#_Toc148100250)

[**Table S13.** Analyses restricted to participants who answered a minimum of 2 WebQs, taken at any point. (N=81,801) 12](#_Toc148100251)

[**Table S14.** Associations between individual components of the Hepatic Steatosis Index and types and sources of dietary carbohydrates, by quintiles of intake. 13](#_Toc148100252)

Figures

[**Figure S1**. Timeline of UK Biobank data collection relevant to this study’s exposures and outcomes. 14](#_Toc148100253)

[**Figure S2**. Types and sources of dietary carbohydrates used as exposures for this study 14](#_Toc148100254)

**Figure S3.** Flowchart of UK Biobank showing exclusion of participants included in the cross-sectional and prospective samples………………………………………………………………………………………...……………..15

| **Table S1.** Data-field codes used to estimate dietary variables | | |
| --- | --- | --- |
| **Total carbohydrates from diet^1^ (g)** | 26013 |  |
| **Free sugar^1^(g)** | 26012 |  |
| **Non-free sugar^1^(g)** | Total sugars (data-field 26011) minus free sugars |  |
| **Starch from refined grains*(g)** |  |  |
|  | 26073 | White bread |
|  | 26113 | White pasta and rice |
|  | 26116 | Pizza |
|  | 26134 | Savoury snacks |
|  | 26128 | Samosa, pakora |
|  | 26085 | Other desserts and cakes and pastries |
|  | 26083 | Savoury crackers |
|  | 26068 | Biscuits |
| **Starch from whole grains*(g)** |  |  |
|  | 26074 | Wholemeal bread |
|  | 26078 | Oat cereal (sugar) |
|  | 26077 | Oat cereal (non sugar) |
|  | 26079 | Other cereal (sugar) |
|  | 26114 | Wholemeal pasta, brown rice and other wholegrains |
|  | 26076 | Bran cereal |
|  | 26105 | Muesli |
|  | 26075 | Biscuit cereal |
|  | 26114 | Wholemeal pasta, brown rice and other wholegrains |
|  | 26097 | Grain dishes - added fat |
| **Total starch from diet(g)** | 26031 |  |
| **Englyst fibre (g)** | 26017 |  |
| **Total energy intake (kJ)** | 26002 |  |
| **Saturated fatty acids^1^ (g)** | 26014 |  |
| **Polyunsaturated fatty acids^1^(g)** | 26015 +26016 |  |
| * Estimated starch provided was calculated for each food group. Variables that provide the nutrients for each of the updated 93 food groups from the UK Biobank, in this case starch, can be obtained upon request.  ^1^After obtaining estimated (g) of intake, intakes were calculated as % of total energy intake. | | |

| **Table S2.** Exclusion criteria | |  |  |
| --- | --- | --- | --- |
| **Exclusion** | **Description** | **Hospital Episode Statistics**  **(ICD10)^1^** | **Self-reported Assessment Centre Data**^2^ |
| **Liver disease** | Any alcoholic liver disease | K70 |  |
|  | Any toxic liver disease | K71 |  |
|  | Hepatic failure, not elsewhere classified | K72 |  |
|  | Chronic hepatitis, not elsewhere classified | K73 |  |
|  | Autoimmune hepatitis | K75.4 |  |
|  | Inflammatory liver disease, unspecified | K75.9 |  |
|  | Liver failure/ cirrhosis |  | Verbal interview |
|  | Infective/viral hepatitis |  |  |
|  | Non-infective hepatitis |  |  |
|  | Hepatitis |  |  |
|  | Acute myocardial infarction | I21 |  |
| **Cardiovascular disease** | Subsequent myocardial infarction | I22 |  |
|  | Complications of myocardial infarction | I23 |  |
|  | Old myocardial infarction | I25.2 |  |
|  | Dressler’s syndrome | I24.1 |  |
|  | Subarachnoid haemorrhage or infarction | I60 |  |
|  | Intracerebral haemorrhage | I61 |  |
|  | Cerebral infarction | I63 |  |
|  | Stroke, not classified as haemorrhage | I64 |  |
|  | Stroke |  | Touch screen^3^/verbal interview |
|  | Heart attack/myocardial infarction |  |  |
|  | Angina |  |  |
|  | Peripheral vascular disease |  | Verbal interview |
|  | Transient ischaemic attack |  |  |
|  | Subdural haemorrhage |  |  |
|  | Aortic aneurysm rupture |  |  |
| **Diabetes** | Diagnosed (or insulin user) |  | Touch screen^4^/verbal interview |
| **Cholesterol lowering medication** |  |  | Touch screen^4^ |
| **Medication** | Dexamethasone |  | Verbal interview |
|  | Hydroprednisone |  |  |
|  | Rifampicin |  |  |
| **Pregnancy** |  |  | Verbal interview |
|  |  |  |  |
| **Endocrine disease** | Thyrotoxicosis |  | Verbal interview |
|  | Cushing syndrome |  |  |
| ^1^ All ICD-10 (International Classification of Diseases and related health problems) diagnoses were obtained from Data field 41270  ^2^All verbal interview diagnoses were obtained from Data-Field 20002: Non-cancer illness, self-reported  ^3^From Data-Field 6150: vascular/heart problems diagnosed by doctor  ^4^ From Data-field 6177 and 6153: medication for cholesterol, blood pressure or diabetes | | | |
|  | | | |

| **Table S3.** Data-fields used for variables included in the analyses | | |
| --- | --- | --- |
|  | **Data-field** | **Additional information** |
| **Outcome** |  |  |
| Alanine aminotransferase | 30620 |  |
| Aspartate aminotransferase | 30650 |  |
| Body mass index (BMI) | 23104 |  |
| Liver PDFF (fat fraction), from the IDEAL protocol | 21088 |  |
| **Confounders** |  |  |
| Sex | 31 |  |
| Age at recruitment | 21022 |  |
| Ethnic background | 21000 |  |
| Qualifications | 6138 | If participants selected more than one, the highest was used to estimate qualification |
| Physical activity |  |  |
|  | *Variables used to calculate total MET hours per week* | |
|  | 864 | Number of days/week walked 10+ minutes |
|  | 874 | Duration of walks |
|  | 884 | Number of days/week of moderate physical activity 10+ minutes |
|  | 894 | Duration of moderate activity |
|  | 904 | Number of days/week of vigorous physical activity 10+ minutes |
|  | 914 | Duration of vigorous activity |
|  | 189 | Townsend deprivation index |
|  | 54 | UK Biobank assessment centre (to calculate region) |
| Smoking status | 20116 |  |
| Alcohol intake |  |  |
|  | *Variables used to calculate total alcohol intake. If only intakes per month were reported, they were divided by 4.3* | |
|  | 1588 | Average weekly beer plus cider intake |
|  |  |  |
|  | 1568 | Average weekly red wine intake |
|  | 4407 | Average monthly red wine intake |
|  | 4429 | Average monthly beer plus cider intake |
|  | 4418 | Average monthly champagne plus white wine intake |
|  | 1578 | Average weekly champagne plus white wine intake |
|  | 1608 | Average weekly fortified wine intake |
|  | 4451 | Average monthly fortified wine intake |
|  | 1598 | Average weekly spirits intake |
|  | 4440 | Average monthly spirits intake |
|  | 5364 | Average weekly intake of other alcoholic drinks |
|  | 4462 | Average monthly intake of other alcoholic drinks |
| High blood pressure |  |  |
|  | 6177 and 6153 | Medication for cholesterol, blood pressure or diabetes |
|  | 20002 | Verbal interview: hypertension |

| **Table S4.** Difference with liver fat geometric mean in quintile 1, presented as percentage (%). Prospective analyses (N=9,268) | | | | | | |
| --- | --- | --- | --- | --- | --- | --- |
|  | **Total carbohydrates** | **Free sugars** | **Non-free sugars** | **Starch from refined grains** | **Starch from whole grains** | **Fibre** |
| Q2 | -4.6 | 0.4 | -5.9 | 3.8 | -3.4 | -6.2 |
| Q3 | -5.7 | 3.5 | -9.7 | 2.8 | -7.1 | -11.3 |
| Q4 | -7.2 | 3.7 | -10.6 | 2.8 | -7.9 | -13.7 |
| Q5 | -6.8 | 5.7 | -12.9 | 3.9 | -10.7 | -16.9 |

| **Table S5**. Cross-sectional analyses (N=22,973) of the odds ratio (OR) and 95% confidence intervals (95%CI) of HSI≥36, by quintiles of intake of carbohydrate types and sources with sequential adjustment for confounders. | | | | | | | | | | | | | | | | | | | | | | |
| --- | --- | --- | --- | --- | --- | --- | --- | --- | --- | --- | --- | --- | --- | --- | --- | --- | --- | --- | --- | --- | --- | --- |
| **Model** | **Q** |  | **Total carbohydrates** | | |  | **Free sugars** | |  | **Non-free sugars** | | |  | **Starch from refined grains** | |  | **Starch from whole grains** | |  | **Fibre** | |  |
|  |  |  | **OR (95%CI)** | | **p** |  | **OR (95%CI)** | **p** |  | **OR (95%CI)** | **p** |  | | **OR (95%CI)** | **p** |  | **OR (95%CI)** | **p** |  | **OR (95%CI)** | **p** |  |
| **+ Age and sex** | **2** |  | 0.89 (0.82-0.97) | 0.009 | |  | 0.95 (0.87-1.04) | 0.290 |  | 0.71 (0.65-0.77) | <0.001 |  | | 1.00 (0.91-1.10) | 0.967 |  | 0.87 (0.80-0.95) | <0.001 |  | 0.82 (0.75-0.89) | <0.001 |  |
|  | **3** |  | 0.82 (0.75-0.89) | <0.001 | |  | 0.89 (0.81-0.98) | 0.010 |  | 0.70 (0.64-0.76) | <0.001 |  | | 1.14 (1.04-1.25) | 0.005 |  | 0.73 (0.67-0.80) | <0.001 |  | 0.66 (0.61-0.73) | <0.001 |  |
|  | **4** |  | 0.82 (0.75-0.89) | <0.001 | |  | 0.95 (0.87-1.04) | 0.230 |  | 0.64 (0.59-0.70) | <0.001 |  | | 1.20 (1.10-1.32) | <0.001 |  | 0.64 (0.58-0.70) | <0.001 |  | 0.59 (0.54-0.65) | <0.001 |  |
|  | **5** |  | 0.76 (0.69-0.83) | <0.001 | |  | 1.10 (1.01-1.20) | 0.040 |  | 0.58 (0.53-0.64) | <0.001 |  | | 1.46 (1.33-1.60) | <0.001 |  | 0.49 (0.45-0.54) | <0.001 |  | 0.51 (0.46-0.56) | <0.001 |  |
| **+ Ethnicity** | **2** |  | 0.89 (0.81-0.97) | 0.008 | |  | 0.95 (0.87-1.04) | 0.290 |  | 0.71 (0.65-0.77) | <0.001 |  | | 1.00 (0.91-1.10) | 0.955 |  | 0.87 (0.80-0.95) | <0.001 |  | 0.82 (0.76-0.90) | <0.001 |  |
|  | **3** |  | 0.82 (0.75-0.89) | <0.001 | |  | 0.89 (0.81-0.98) | 0.010 |  | 0.70 (0.64-0.77) | <0.001 |  | | 1.14 (1.04-1.25) | 0.004 |  | 0.73 (0.67-0.80) | <0.001 |  | 0.67 (0.61-0.73) | <0.001 |  |
|  | **4** |  | 0.81 (0.75-0.89) | <0.001 | |  | 0.94 (0.86-1.03) | 0.200 |  | 0.65 (0.59-0.71) | <0.001 |  | | 1.20 (1.10-1.32) | <0.001 |  | 0.64 (0.58-0.70) | <0.001 |  | 0.60 (0.55-0.65) | <0.001 |  |
|  | **5** |  | 0.75 (0.69-0.82) | <0.001 | |  | 1.08 (0.99-1.18) | 0.080 |  | 0.58 (0.53-0.64) | <0.001 |  | | 1.46 (1.34-1.60) | <0.001 |  | 0.49 (0.45-0.54) | <0.001 |  | 0.51 (0.47-0.56) | <0.001 |  |
| **+Deprivation, education, and region** | **2** |  | 0.88 (0.80-0.96) | 0.003 | |  | 0.95 (0.87-1.04) | 0.280 |  | 0.72 (0.66-0.79) | <0.001 |  | | 1.00 (0.91-1.09) | 0.949 |  | 0.87 (0.80-0.95) | <0.001 |  | 0.84 (0.77-0.91) | <0.001 |  |
|  | **3** |  | 0.79 (0.73-0.87) | <0.001 | |  | 0.89 (0.81-0.97) | 0.010 |  | 0.71 (0.65-0.77) | <0.001 |  | | 1.13 (1.03-1.24) | 0.008 |  | 0.75 (0.68-0.82) | <0.001 |  | 0.69 (0.63-0.75) | <0.001 |  |
|  | **4** |  | 0.78 (0.71-0.85) | <0.001 | |  | 0.93 (0.85-1.02) | 0.140 |  | 0.65 (0.59-0.71) | <0.001 |  | | 1.19 (1.09-1.31) | <0.001 |  | 0.65 (0.59-0.71) | <0.001 |  | 0.62 (0.57-0.68) | <0.001 |  |
|  | **5** |  | 0.71 (0.64-0.77) | <0.001 | |  | 1.05 (0.96-1.15) | 0.290 |  | 0.58 (0.53-0.64) | <0.001 |  | | 1.43 (1.30-1.56) | <0.001 |  | 0.50 (0.45-0.54) | <0.001 |  | 0.53 (0.48-0.58) | <0.001 |  |
| **+ Smoking status** | **2** |  | 0.89 (0.81-0.97) | 0.008 | |  | 0.96 (0.88-1.05) | 0.390 |  | 0.72 (0.66-0.79) | <0.001 |  | | 1.00 (0.91-1.10) | 0.982 |  | 0.87 (0.80-0.95) | <0.001 |  | 0.84 (0.77-0.92) | <0.001 |  |
|  | **3** |  | 0.81 (0.74-0.88) | <0.001 | |  | 0.90 (0.82-0.98) | 0.020 |  | 0.72 (0.66-0.78) | <0.001 |  | | 1.13 (1.03-1.24) | 0.008 |  | 0.75 (0.69-0.82) | <0.001 |  | 0.69 (0.63-0.76) | <0.001 |  |
|  | **4** |  | 0.79 (0.72-0.87) | <0.001 | |  | 0.94 (0.86-1.03) | 0.210 |  | 0.66 (0.60-0.72) | <0.001 |  | | 1.19 (1.09-1.31) | <0.001 |  | 0.65 (0.60-0.71) | <0.001 |  | 0.62 (0.57-0.68) | <0.001 |  |
|  | **5** |  | 0.72 (0.66-0.79) | <0.001 | |  | 1.06 (0.97-1.16) | 0.220 |  | 0.59 (0.53-0.65) | <0.001 |  | | 1.43 (1.30-1.57) | <0.001 |  | 0.50 (0.46-0.55) | <0.001 |  | 0.53 (0.48-0.58) | <0.001 |  |
| **+ Physical activity** | **2** |  | 0.89 (0.82-0.98) | 0.012 | |  | 0.96 (0.88-1.05) | 0.390 |  | 0.73 (0.67-0.80) | <0.001 |  | | 0.99 (0.90-1.09) | 0.823 |  | 0.88 (0.81-0.96) | <0.001 |  | 0.85 (0.78-0.92) | <0.001 |  |
|  | **3** |  | 0.82 (0.75-0.89) | <0.001 | |  | 0.89 (0.82-0.98) | 0.020 |  | 0.74 (0.67-0.81) | <0.001 |  | | 1.12 (1.02-1.23) | 0.017 |  | 0.75 (0.69-0.82) | <0.001 |  | 0.70 (0.64-0.77) | <0.001 |  |
|  | **4** |  | 0.82 (0.75-0.90) | <0.001 | |  | 0.95 (0.87-1.04) | 0.250 |  | 0.69 (0.63-0.76) | <0.001 |  | | 1.16 (1.06-1.28) | 0.001 |  | 0.66 (0.60-0.72) | <0.001 |  | 0.64 (0.58-0.70) | <0.001 |  |
|  | **5** |  | 0.75 (0.69-0.82) | <0.001 | |  | 1.06 (0.97-1.16) | 0.190 |  | 0.63 (0.57-0.69) | <0.001 |  | | 1.38 (1.26-1.51) | <0.001 |  | 0.51 (0.46-0.56) | <0.001 |  | 0.56 (0.51-0.62) | <0.001 |  |
| **+ Alcohol intake** | **2** |  | 0.87 (0.80-0.95) | 0.002 | |  | 0.96 (0.88-1.05) | 0.400 |  | 0.73 (0.67-0.80) | <0.001 |  | | 0.99 (0.90-1.09) | 0.834 |  | 0.88 (0.81-0.96) | <0.001 |  | 0.85 (0.78-0.92) | <0.001 |  |
|  | **3** |  | 0.78 (0.71-0.86) | <0.001 | |  | 0.90 (0.82-0.98) | 0.020 |  | 0.73 (0.67-0.80) | <0.001 |  | | 1.12 (1.02-1.22) | 0.019 |  | 0.75 (0.69-0.82) | <0.001 |  | 0.70 (0.64-0.77) | <0.001 |  |
|  | **4** |  | 0.77 (0.70-0.85) | <0.001 | |  | 0.94 (0.86-1.03) | 0.220 |  | 0.68 (0.62-0.75) | <0.001 |  | | 1.16 (1.06-1.27) | 0.002 |  | 0.66 (0.60-0.72) | <0.001 |  | 0.64 (0.58-0.70) | <0.001 |  |
| **+ Total energy intake** | **2** |  | 0.87 (0.79-0.95) | 0.002 | |  | 0.96 (0.88-1.05) | 0.400 |  | 0.74 (0.68-0.81) | <0.001 |  | | 0.99 (0.90-1.09) | 0.815 |  | 0.89 (0.82-0.97) | <0.001 |  | 0.81 (0.74-0.89) | <0.001 |  |
|  | **3** |  | 0.78 (0.71-0.86) | <0.001 | |  | 0.89 (0.81-0.98) | 0.020 |  | 0.74 (0.68-0.82) | <0.001 |  | | 1.11 (1.01-1.22) | 0.026 |  | 0.77 (0.70-0.84) | <0.001 |  | 0.65 (0.59-0.72) | <0.001 |  |
|  | **4** |  | 0.77 (0.70-0.84) | <0.001 | |  | 0.94 (0.85-1.03) | 0.170 |  | 0.70 (0.63-0.76) | <0.001 |  | | 1.16 (1.05-1.27) | 0.002 |  | 0.67 (0.61-0.73) | <0.001 |  | 0.57 (0.51-0.62) | <0.001 |  |
|  | **5** |  | 0.68 (0.62-0.75) | <0.001 | |  | 1.03 (0.94-1.13) | 0.560 |  | 0.62 (0.56-0.68) | <0.001 |  | | 1.35 (1.23-1.48) | <0.001 |  | 0.51 (0.47-0.56) | <0.001 |  | 0.46 (0.41-0.51) | <0.001 |  |
| **+PUFA/SFA ratio** | **2** |  | 0.87 (0.80-0.95) | <0.001 | |  | 0.95 (0.86-1.04) | 0.250 |  | 0.74 (0.68-0.81) | <0.001 |  | | 0.98 (0.89-1.07) | 0.612 |  | 0.89 (0.82-0.98) | <0.001 |  | 0.81 (0.74-0.89) | <0.001 |  |
|  | **3** |  | 0.79 (0.72-0.86) | <0.001 | |  | 0.87 (0.79-0.96) | 0.000 |  | 0.75 (0.69-0.82) | <0.001 |  | | 1.09 (1.00-1.20) | 0.060 |  | 0.77 (0.71-0.85) | <0.001 |  | 0.65 (0.59-0.72) | <0.001 |  |
|  | **4** |  | 0.78 (0.71-0.85) | <0.001 | |  | 0.91 (0.83-1.00) | 0.050 |  | 0.70 (0.64-0.77) | <0.001 |  | | 1.14 (1.03-1.25) | 0.007 |  | 0.68 (0.62-0.74) | <0.001 |  | 0.57 (0.51-0.63) | <0.001 |  |
|  | **5** |  | 0.70 (0.64-0.77) | <0.001 | |  | 0.99 (0.90-1.09) | 0.830 |  | 0.63 (0.57-0.70) | <0.001 |  | | 1.33 (1.21-1.46) | <0.001 |  | 0.52 (0.47-0.57) | <0.001 |  | 0.46 (0.41-0.52) | <0.001 |  |

| **Table S6.** Sequential adjustment for confounders (N=9,268). Difference with geometric mean of liver fat in Q1 of types and sources of carbohydrates, obtained from linear regression models. | | | | | | | |
| --- | --- | --- | --- | --- | --- | --- | --- |
| **Model** |  | **Total Cho** | **Free sugars** | **Non-free sugars** | **Starch from refined grains** | **Starch from whole grains** | **Fibre (g)** |
| **Age and sex** | Q2 | -0.18 (-0.30,-0.05) | -0.06 (-0.18,0.05) | -0.32 (-0.44,-0.19) | 0.13 (0.01,0.25) | -0.15 (-0.28,-0.03) | -0.22 (-0.35,-0.09) |
|  | Q3 | -0.24 (-0.37,-0.12) | 0.03 (-0.09,0.15) | -0.38 (-0.51,-0.26) | 0.11 (0.00,0.23) | -0.33 (-0.45,-0.20) | -0.40 (-0.52,-0.27) |
|  | Q4 | -0.25 (-0.37,-0.13) | 0.09 (-0.03,0.21) | -0.46 (-0.59,-0.34) | 0.18 (0.06,0.30) | -0.39 (-0.52,-0.27) | -0.47 (-0.59,-0.35) |
|  | Q5 | -0.27 (-0.40,-0.15) | 0.16 (0.03,0.28) | -0.51 (-0.63,-0.38) | 0.22 (0.10,0.34) | -0.53 (-0.65,-0.41) | -0.54 (-0.66,-0.42) |
| **+ Ethnicity** | Q2 | -0.18 (-0.30,-0.05) | -0.07 (-0.19,0.05) | -0.31 (-0.44,-0.19) | 0.13 (0.01,0.25) | -0.16 (-0.29,-0.03) | -0.23 (-0.36,-0.10) |
|  | Q3 | -0.24 (-0.36,-0.12) | 0.03 (-0.09,0.15) | -0.38 (-0.51,-0.26) | 0.11 (0.00,0.23) | -0.33 (-0.46,-0.21) | -0.40 (-0.53,-0.28) |
|  | Q4 | -0.25 (-0.37,-0.13) | 0.09 (-0.03,0.21) | -0.46 (-0.59,-0.33) | 0.18 (0.07,0.30) | -0.40 (-0.52,-0.28) | -0.48 (-0.60,-0.36) |
|  | Q5 | -0.27 (-0.40,-0.15) | 0.16 (0.04,0.28) | -0.51 (-0.63,-0.38) | 0.22 (0.10,0.34) | -0.53 (-0.65,-0.41) | -0.55 (-0.67,-0.42) |
| **+SES** | Q2 | -0.20 (-0.33,-0.08) | -0.06 (-0.17,0.06) | -0.30 (-0.43,-0.18) | 0.13 (0.01,0.25) | -0.16 (-0.28,-0.03) | -0.22 (-0.34,-0.09) |
|  | Q3 | -0.27 (-0.40,-0.15) | 0.03 (-0.09,0.15) | -0.36 (-0.49,-0.23) | 0.11 (-0.01,0.23) | -0.32 (-0.45,-0.20) | -0.38 (-0.51,-0.26) |
|  | Q4 | -0.29 (-0.41,-0.17) | 0.08 (-0.04,0.20) | -0.43 (-0.56,-0.31) | 0.17 (0.05,0.29) | -0.39 (-0.51,-0.26) | -0.45 (-0.58,-0.33) |
|  | Q5 | -0.32 (-0.44,-0.20) | 0.14 (0.02,0.26) | -0.48 (-0.61,-0.36) | 0.19 (0.07,0.31) | -0.53 (-0.65,-0.41) | -0.53 (-0.65,-0.40) |
| **+ Smoking status** | Q2 | -0.20 (-0.32,-0.08) | -0.05 (-0.17,0.06) | -0.30 (-0.43,-0.17) | 0.13 (0.01,0.25) | -0.15 (-0.28,-0.02) | -0.21 (-0.34,-0.08) |
|  | Q3 | -0.27 (-0.39,-0.14) | 0.03 (-0.09,0.15) | -0.35 (-0.48,-0.23) | 0.11 (-0.01,0.23) | -0.32 (-0.44,-0.19) | -0.38 (-0.50,-0.25) |
|  | Q4 | -0.29 (-0.41,-0.16) | 0.08 (-0.04,0.20) | -0.42 (-0.55,-0.30) | 0.17 (0.05,0.29) | -0.38 (-0.50,-0.26) | -0.45 (-0.57,-0.32) |
|  | Q5 | -0.32 (-0.44,-0.20) | 0.14 (0.02,0.26) | -0.48 (-0.60,-0.35) | 0.19 (0.07,0.31) | -0.52 (-0.64,-0.40) | -0.52 (-0.64,-0.40) |
| **+ Physical activity** | Q2 | -0.20 (-0.32,-0.08) | -0.05 (-0.17,0.06) | -0.31 (-0.44,-0.18) | 0.13 (0.01,0.25) | -0.15 (-0.28,-0.02) | -0.22 (-0.35,-0.09) |
|  | Q3 | -0.26 (-0.38,-0.13) | 0.04 (-0.08,0.15) | -0.35 (-0.48,-0.23) | 0.11 (0.00,0.23) | -0.31 (-0.44,-0.19) | -0.38 (-0.51,-0.26) |
|  | Q4 | -0.27 (-0.39,-0.14) | 0.07 (-0.04,0.19) | -0.42 (-0.54,-0.29) | 0.16 (0.04,0.28) | -0.38 (-0.51,-0.26) | -0.45 (-0.57,-0.33) |
|  | Q5 | -0.30 (-0.42,-0.18) | 0.15 (0.03,0.27) | -0.47 (-0.59,-0.34) | 0.18 (0.06,0.30) | -0.51 (-0.63,-0.39) | -0.51 (-0.63,-0.39) |
| **+ Alcohol intake** | Q2 | -0.21 (-0.33,-0.08) | -0.05 (-0.17,0.07) | -0.31 (-0.44,-0.18) | 0.13 (0.02,0.25) | -0.15 (-0.27,-0.02) | -0.22 (-0.35,-0.09) |
|  | Q3 | -0.28 (-0.40,-0.15) | 0.04 (-0.08,0.16) | -0.36 (-0.49,-0.23) | 0.12 (0.00,0.23) | -0.31 (-0.43,-0.18) | -0.38 (-0.51,-0.26) |
|  | Q4 | -0.30 (-0.43,-0.18) | 0.07 (-0.05,0.19) | -0.43 (-0.56,-0.30) | 0.16 (0.04,0.28) | -0.38 (-0.50,-0.26) | -0.45 (-0.58,-0.33) |
|  | Q5 | -0.36 (-0.48,-0.23) | 0.14 (0.02,0.27) | -0.49 (-0.62,-0.36) | 0.18 (0.06,0.30) | -0.52 (-0.64,-0.40) | -0.52 (-0.64,-0.40) |
| **+ Total energy intake** | Q2 | -0.21 (-0.34,-0.09) | -0.06 (-0.17,0.06) | -0.29 (-0.42,-0.16) | 0.13 (0.01,0.25) | -0.14 (-0.27,-0.01) | -0.30 (-0.43,-0.16) |
|  | Q3 | -0.28 (-0.41,-0.16) | 0.03 (-0.09,0.15) | -0.34 (-0.47,-0.21) | 0.11 (-0.01,0.23) | -0.30 (-0.43,-0.18) | -0.49 (-0.62,-0.35) |
|  | Q4 | -0.31 (-0.43,-0.19) | 0.06 (-0.06,0.18) | -0.41 (-0.53,-0.28) | 0.15 (0.03,0.27) | -0.37 (-0.49,-0.24) | -0.59 (-0.72,-0.45) |
|  | Q5 | -0.35 (-0.48,-0.23) | 0.13 (0.00,0.25) | -0.46 (-0.59,-0.34) | 0.16 (0.05,0.28) | -0.51 (-0.63,-0.39) | -0.70 (-0.83,-0.57) |
| **+ BMI** | Q2 | -0.14 (-0.25,-0.02) | 0.03 (-0.08,0.14) | -0.19 (-0.31,-0.07) | 0.12 (0.01,0.23) | -0.11 (-0.22,0.01) | -0.22 (-0.34,-0.09) |
|  | Q3 | -0.17 (-0.29,-0.06) | 0.12 (0.01,0.23) | -0.29 (-0.41,-0.18) | 0.10 (-0.01,0.21) | -0.22 (-0.34,-0.11) | -0.37 (-0.49,-0.25) |
|  | Q4 | -0.21 (-0.33,-0.10) | 0.14 (0.03,0.25) | -0.32 (-0.44,-0.20) | 0.10 (-0.01,0.21) | -0.24 (-0.35,-0.13) | -0.43 (-0.55,-0.31) |
|  | Q5 | -0.23 (-0.34,-0.11) | 0.21 (0.10,0.32) | -0.40 (-0.52,-0.29) | 0.13 (0.02,0.24) | -0.34 (-0.45,-0.23) | -0.54 (-0.66,-0.41) |
| **+PUFA/SFA ratio** | Q2 | -0.14 (-0.25,-0.02) | 0.01 (-0.10,0.12) | -0.19 (-0.31,-0.06) | 0.11 (0.00,0.22) | -0.11 (-0.22,0.01) | -0.20 (-0.33,-0.07) |
|  | Q3 | -0.16 (-0.28,-0.05) | 0.10 (-0.01,0.21) | -0.29 (-0.41,-0.17) | 0.08 (-0.02,0.19) | -0.21 (-0.33,-0.10) | -0.34 (-0.46,-0.21) |
|  | Q4 | -0.20 (-0.32,-0.09) | 0.11 (0.00,0.22) | -0.30 (-0.42,-0.19) | 0.08 (-0.03,0.19) | -0.23 (-0.34,-0.11) | -0.39 (-0.52,-0.27) |
|  | Q5 | -0.19 (-0.31,-0.08) | 0.17 (0.05,0.28) | -0.37 (-0.49,-0.25) | 0.11 (0.00,0.22) | -0.31 (-0.42,-0.19) | -0.48 (-0.60,-0.35) |
| SES: socioeconomic status, BMI: body mass index | | | | | | | |

| **Table S7.** Test for heterogeneity by sex | | | |
| --- | --- | --- | --- |
| **Nutrient (continuous)** | **Cross-sectional analyses**  **N=22,973** |  | **Prospective analyses**  **N=9,268** |
|  | **Heterogeneity X^2^_(1)_** |  | **Heterogeneity X^2^_(1)_** |
| **Total carbohydrates** | 1.3 (p=0.25) |  | 0.7 (p=0.39) |
| **Free sugar** | 0.5 (p=0.46) |  | 0.006 (p=0.94) |
| **Non free sugar** | 0.3 (p=0.57) |  | 1.3 (p=0.26) |
| **Starch from refined grains** | 2.2 (p=0.13) |  | 0.1 (p=0.81) |
| **Starch from whole grains** | 0.1 (p=0.70) |  | 3.8 (p=0.05) |
| **Fibre** | 1.0 (p=0.32) |  | 0.2 (p=0.63) |

| **Table S8**. Test for heterogeneity across groups of Body Mass Index (BMI). Estimated change in mean liver fat percentage per 1% increase of nutrient, by groups of BMI, obtained from fully adjusted linear regression models. | | | | | | |
| --- | --- | --- | --- | --- | --- | --- |
|  | **Total carbohydrates** | **Free sugars** | **Non-free sugars** | **Starch from refined grains** | **Starch from whole grains** | **Fibre** |
| **BMI -25** | -0.34 (-0.60, -0.09) | 0.46 (0.08,0.84) | -0.40 (-0.72, -0.08) | -0.10 (-0.37,0.18) | -0.70 (-1.09, -0.31) | -0.92 (-1.26, -0.58) |
| **BMI 25-** | -0.36 (-0.62, -0.09) | 0.42 (0.03,0.82) | -0.92 (-1.27, -0.57) | 0.39 (0.08,0.71) | -1.01 (-1.48, -0.54) | -0.83 (-1.20, -0.46) |
| **BMI 30-** | -0.32 (-0.76,0.12) | 1.08 (0.41,1.75) | -1.67 (-2.25, -1.09) | 0.73 (0.20,1.27) | -1.01 (-1.86, -0.16) | -1.23 (-1.81, -0.64) |
| **p heterogeneity** | 0.99 | 0.22 | <0.001 | 0.01 | 0.57 | 0.50 |

| **Table S9**. Sensitivity analyses of participants who answered at least four 24-hr dietary assessments in cross-sectional analyses (N=9,046). ORs of HSI≥36, by quintiles of carbohydrates types and sources. Results from logistic regression models controlling for age, sex, ethnicity, deprivation, education, region, smoking status, physical activity, alcohol intake, total energy intake and PUFA:SFA ratio. | | | | | | | | | | | | |
| --- | --- | --- | --- | --- | --- | --- | --- | --- | --- | --- | --- | --- |
|  |  | **Total Carbohydrates** |  | **Free sugars** |  | **Non-free sugars** |  | **Starch from refined grains** |  | **Starch from whole grains** |  | **Fibre** |
| **Q2** |  | 0.87 (0.82,0.93) |  | 0.89 (0.80,0.99) |  | 0.71 (0.64,0.79) |  | 0.97 (0.87,1.08) |  | 0.86 (0.78,0.96) |  | 0.68 (0.61,0.75) |
| **Q3** |  | 0.79 (0.74,0.84) |  | 0.82 (0.74,0.91) |  | 0.68 (0.61,0.75) |  | 1.20 (1.08,1.33) |  | 0.72 (0.65,0.80) |  | 0.57 (0.51,0.63) |
| **Q4** |  | 0.78 (0.73,0.83) |  | 0.84 (0.76,0.94) |  | 0.65 (0.58,0.72) |  | 1.16 (1.04,1.28) |  | 0.66 (0.60,0.74) |  | 0.53 (0.47,0.59) |
| **Q5** | | 0.70 (0.65,0.75) |  | 0.93 (0.84,1.04) |  | 0.56 (0.49,0.63) |  | 1.33 (1.19,1.47) |  | 0.43 (0.38,0.49) |  | 0.35 (0.31,0.41) |

| **Table S10.** Sensitivity analyses of participants who answered at least four 24-hr dietary assessments in prospective analyses (N=2,829). Difference with geometric mean of liver fat in Q1 of types and sources of carbohydrates, obtained from linear regression models controlling for age, sex, ethnicity, deprivation, education, region, smoking status, physical activity, alcohol intake, total energy intake and PUFA:SFA ratio. | | | | | | |
| --- | --- | --- | --- | --- | --- | --- |
| **Difference with Q1** | **Total Cho** | **Free sugars** | **Non-free sugars** | **Starch from refined grains** | **Starch from whole grains** | **Fibre** |
| Q2 | -0.13 (-0.34,0.07) | -0.10 (-0.29,0.08) | -0.36 (-0.58, -0.14) | 0.13 (-0.06,0.32) | -0.12 (-0.33,0.10) | -0.14 (-0.38,0.09) |
| Q3 | -0.16 (-0.37,0.05) | 0.16 (-0.05,0.37) | -0.36 (-0.58, -0.15) | 0.05 (-0.14,0.23) | -0.28 (-0.48, -0.09) | -0.36 (-0.59, -0.13) |
| Q4 | -0.32 (-0.52, -0.11) | -0.02 (-0.22,0.17) | -0.43 (-0.65, -0.21) | 0.21 (0.01,0.41) | -0.24 (-0.45, -0.04) | -0.42 (-0.64, -0.19) |
| Q5 | -0.23 (-0.44, -0.03) | 0.11 (-0.09,0.31) | -0.50 (-0.72, -0.29) | 0.13 (-0.06,0.33) | -0.30 (-0.51, -0.10) | -0.56 (-0.79, -0.33) |

| **Table S11.** Cross sectional analyses (N=22,793), with additional adjustment for diagnosed high blood pressure^^^. Odds ratio of HSI>36 (95%CI) | | | | | | | | | | | |
| --- | --- | --- | --- | --- | --- | --- | --- | --- | --- | --- | --- |
|  | **Total carbohydrates** |  | **Free sugars** |  | **Non-free sugars** |  | **Starch from refined grains** |  | **Starch from whole grains** |  | **Fibre** |
| **Q2** | 0.88 (0.80,0.96) |  | 0.96 (0.87,1.05) |  | 0.75 (0.68,0.82) |  | 0.97 (0.88,1.07) |  | 0.91 (0.83,0.99) |  | 0.81 (0.74,0.89) |
| **Q3** | 0.78 (0.71,0.86) |  | 0.88 (0.80,0.97) |  | 0.77 (0.70,0.84) |  | 1.09 (0.99,1.19) |  | 0.79 (0.73,0.87) |  | 0.65 (0.59,0.72) |
| **Q4** | 0.78 (0.71,0.85) |  | 0.91 (0.83,1.00) |  | 0.72 (0.65,0.79) |  | 1.13 (1.03,1.24) |  | 0.69 (0.63,0.75) |  | 0.57 (0.52,0.63) |
| **Q5** | 0.69 (0.63,0.76) |  | 0.99 (0.91,1.09) |  | 0.63 (0.57,0.69) |  | 1.31 (1.19,1.44) |  | 0.53 (0.48,0.58) |  | 0.47 (0.42,0.52) |

^High blood pressure was defined as being diagnosed by a doctor (verbal interview), and/or taking blood pressure lowering medication

| **Table S12.** Prospective analyses (N=9,268), with additional adjustment for diagnosed high blood pressure^^^. Difference with liver fat geometric mean in quintile 1 (95%CI) | | | | | | | | | | | |
| --- | --- | --- | --- | --- | --- | --- | --- | --- | --- | --- | --- |
|  | **Total carbohydrates** |  | **Free sugars** |  | **Non-free sugars** |  | **Starch from refined grains** |  | **Starch from whole grains** |  | **Fibre** |
| **Q2** | -0.20 (-0.33, -0.08) |  | -0.08 (-0.20, 0.04) |  | -0.27 (-0.40, -0.14) |  | 0.13 (0.01, 0.24) |  | -0.14 (-0.27, -0.02) |  | -0.28 (-0.41, -0.14) |
| **Q3** | -0.27 (-0.40, -0.15) |  | 0.02 (-0.10, 0.14) |  | -0.34 (-0.46, -0.21) |  | 0.09 (-0.03, 0.21) |  | -0.29 (-0.41, -0.16) |  | -0.45 (-0.58, -0.31) |
| **Q4** | -0.30 (-0.42, -0.17) |  | 0.03 (-0.09, 0.15) |  | -0.38 (-0.50, -0.25) |  | 0.13 (0.01, 0.25) |  | -0.34 (-0.46, -0.22) |  | -0.54 (-0.67, -0.41) |
| **Q5** | -0.31 (-0.43, -0.19) |  | 0.08 (-0.04, 0.20) |  | -0.43 (-0.56, -0.30) |  | 0.15 (0.04, 0.27) |  | -0.45 (-0.57, -0.33) |  | -0.64 (-0.77, -0.50) |

^High blood pressure was defined as being diagnosed by a doctor (verbal interview), and/or taking blood pressure lowering medication

| **Table S13.** Analyses restricted to participants who answered a minimum of 2 WebQs, taken at any point. (N=81,801) | | |
| --- | --- | --- |
| **Nutrient (% of total energy by quintile)** | **OR of HSI≥36** | **p value** |
| **Total carbohydrates** |  |  |
| Q2 (45-49) | 0.88 (0.84-0.93) | 0.000 |
| Q3 (-52.9) | 0.79 (0.75-0.83) | 0.000 |
| Q4 (-56.9) | 0.77 (0.73-0.81) | 0.000 |
| Q5 (-84.1) | 0.71 (0.67-0.74) | 0.000 |
| **Free Sugars** |  |  |
| Q2 (7.5-10.0) | 0.91 (0.87-0.95) | 0.000 |
| Q3 (-12.3) | 0.86 (0.82-0.90) | 0.000 |
| Q4 (-15.4) | 0.85 (0.81-0.90) | 0.000 |
| Q5 (-60) | 0.89 (0.85-0.93) | 0.000 |
| **Non-free sugars** |  |  |
| Q2 (8.8-11.5) | 0.79 (0.75-0.82) | 0.000 |
| Q3 (-14.2) | 0.77 (0.74-0.81) | 0.000 |
| Q4 (-17.9) | 0.75 (0.71-0.79) | 0.000 |
| Q5 (-62.9) | 0.73 (0.69-0.77) | 0.000 |
| **Starch from refined grains** | |  |
| Q2 (6.4-9.8) | 1.01 (0.96-1.06) | 0.709 |
| Q3 (13.0) | 1.05 (1.00-1.10) | 0.050 |
| Q4 (-17.1) | 1.11 (1.06-1.16) | 0.000 |
| Q5 (-50.3) | 1.23 (1.17-1.29) | 0.000 |
| **Starch from whole grains** | |  |
| Q2 (1.7-3.9) | 0.90 (0.86-0.94) | 0.000 |
| Q3 (-6.0) | 0.77 (0.74-0.81) | 0.000 |
| Q4 (-8.7) | 0.67 (0.64-0.70) | 0.000 |
| Q5 (-51.4) | 0.57 (0.54-0.60) | 0.000 |
| **Fibre** |  |  |
| Q2 (13-16g) | 0.85 (0.81-0.89) | 0.000 |
| Q3 (-19g) | 0.73 (0.69-0.77) | 0.000 |
| Q4 (-22g) | 0.65 (0.62-0.69) | 0.000 |
| Q5 (-85g) | 0.55 (0.52-0.58) | 0.000 |
|  |  |  |

| **Table S14.** Associations between individual components of the Hepatic Steatosis Index and types and sources of dietary carbohydrates, by quintiles of intake. | | | | | | | |
| --- | --- | --- | --- | --- | --- | --- | --- |
|  |  | **Total carbohydrates** | **Free sugars** | **Non-free sugars** | **Starch from refined grains** | **Starch from whole grains** | **Fibre** |
| Enzyme ratio^1^ | Q2 | -0.016 (-0.028,-0.005, p value=0.00) | -0.004 (-0.015,0.008, p value=0.54) | -0.049 (-0.060,-0.037, p value=0.00) | -0.006 (-0.017,0.005, p value=0.31) | -0.020 (-0.032,-0.009, p value=0.00) | -0.024 (-0.035,-0.013, p value=0.00) |
|  | Q3 | -0.023 (-0.034,-0.012, p value=0.00) | 0.008 (-0.004,0.019, p value=0.19) | -0.057 (-0.069,-0.046, p value=0.00) | 0.003 (-0.008,0.015, p value=0.55) | -0.027 (-0.038,-0.015, p value=0.00) | -0.039 (-0.051,-0.028, p value=0.00) |
|  | Q4 | -0.041 (-0.052,-0.030, p value=0.00) | 0.012 (0.001,0.024, p value=0.03) | -0.081 (-0.093,-0.070, p value=0.00) | 0.022 (0.011,0.033, p value=0.00) | -0.043 (-0.055,-0.032, p value=0.00) | -0.043 (-0.054,-0.031, p value=0.00) |
|  | Q5 | -0.051 (-0.063,-0.040, p value=0.00) | 0.028 (0.016,0.039, p value=0.00) | -0.113 (-0.124,-0.102, p value=0.00) | 0.036 (0.025,0.047, p value=0.00) | -0.057 (-0.068,-0.045, p value=0.00) | -0.049 (-0.061,-0.038, p value=0.00) |
| BMI | Q2 | -0.3 (-0.5,-0.1, p value=0.00) | -0.2 (-0.4,0.0, p value=0.05) | -0.7 (-0.9,-0.6, p value=0.00) | 0.0 (-0.2,0.2, p value=0.88) | -0.3 (-0.4,-0.1, p value=0.00) | -0.3 (-0.5,-0.1, p value=0.00) |
|  | Q3 | -0.4 (-0.6,-0.2, p value=0.00) | -0.2 (-0.4,0.0, p value=0.03) | -0.7 (-0.9,-0.6, p value=0.00) | 0.3 (0.1,0.4, p value=0.01) | -0.7 (-0.8,-0.5, p value=0.00) | -0.8 (-1.0,-0.6, p value=0.00) |
|  | Q4 | -0.5 (-0.7,-0.3, p value=0.00) | -0.1 (-0.3,0.1, p value=0.34) | -1.1 (-1.3,-0.9, p value=0.00) | 0.3 (0.1,0.5, p value=0.00) | -1.0 (-1.2,-0.8, p value=0.00) | -1.1 (-1.2,-0.9, p value=0.00) |
|  | Q5 | -0.5 (-0.7,-0.3, p value=0.00) | 0.3 (0.1,0.5, p value=0.00) | -1.1 (-1.3,-0.9, p value=0.00) | 0.8 (0.6,1.0, p value=0.00) | -1.4 (-1.6,-1.2, p value=0.00) | -1.3 (-1.4,-1.1, p value=0.00) |
| Sex^3^ | Q2 | 0.99 (0.91,1.074, p value=0.783) | 1.19 (1.09,1.303, p value=0.000) | 0.65 (0.60,0.705, p value=0.000) | 0.91 (0.83,0.986, p value=0.023) | 0.89 (0.82,0.973, p value=0.010) | 0.96 (0.88,1.049, p value=0.380) |
|  | Q3 | 0.85 (0.79,0.929, p value=0.000) | 1.53 (1.40,1.666, p value=0.000) | 0.49 (0.45,0.536, p value=0.000) | 0.98 (0.90,1.071, p value=0.720) | 0.87 (0.80,0.951, p value=0.002) | 1.11 (1.02,1.206, p value=0.019) |
|  | Q4 | 0.84 (0.77,0.915, p value=0.000) | 1.63 (1.50,1.781, p value=0.000) | 0.35 (0.32,0.385, p value=0.000) | 1.07 (0.98,1.162, p value=0.123) | 0.86 (0.79,0.936, p value=0.000) | 1.18 (1.09,1.288, p value=0.000) |
|  | Q5 | 0.80 (0.74,0.872, p value=0.000) | 2.09 (1.92,2.278, p value=0.000) | 0.23 (0.21,0.255, p value=0.000) | 1.19 (1.09,1.293, p value=0.000) | 1.06 (0.98,1.153, p value=0.165) | 1.61 (1.48,1.755, p value=0.000) |
| ^1,2^ Results from crude linear regression models  ^3^ Results from crude logistic regression models (OR of being male, 95%CI) | | | | | | | |


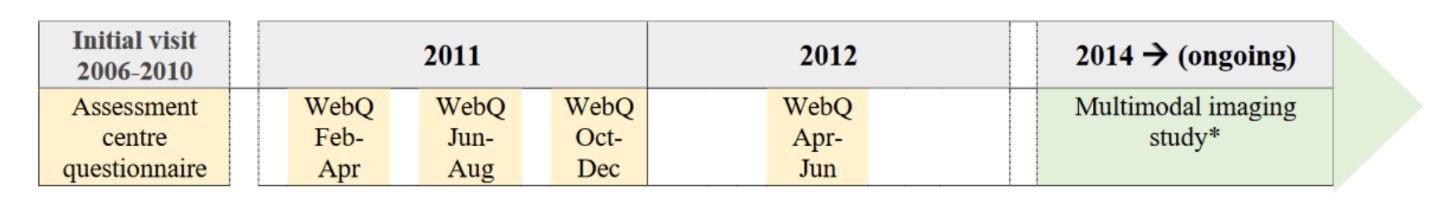


**Figure S1**. Timeline of UK Biobank data collection relevant to this study’s exposures and outcomes. Mean time between last WebQ and liver MRI was 6 years and 3 months.


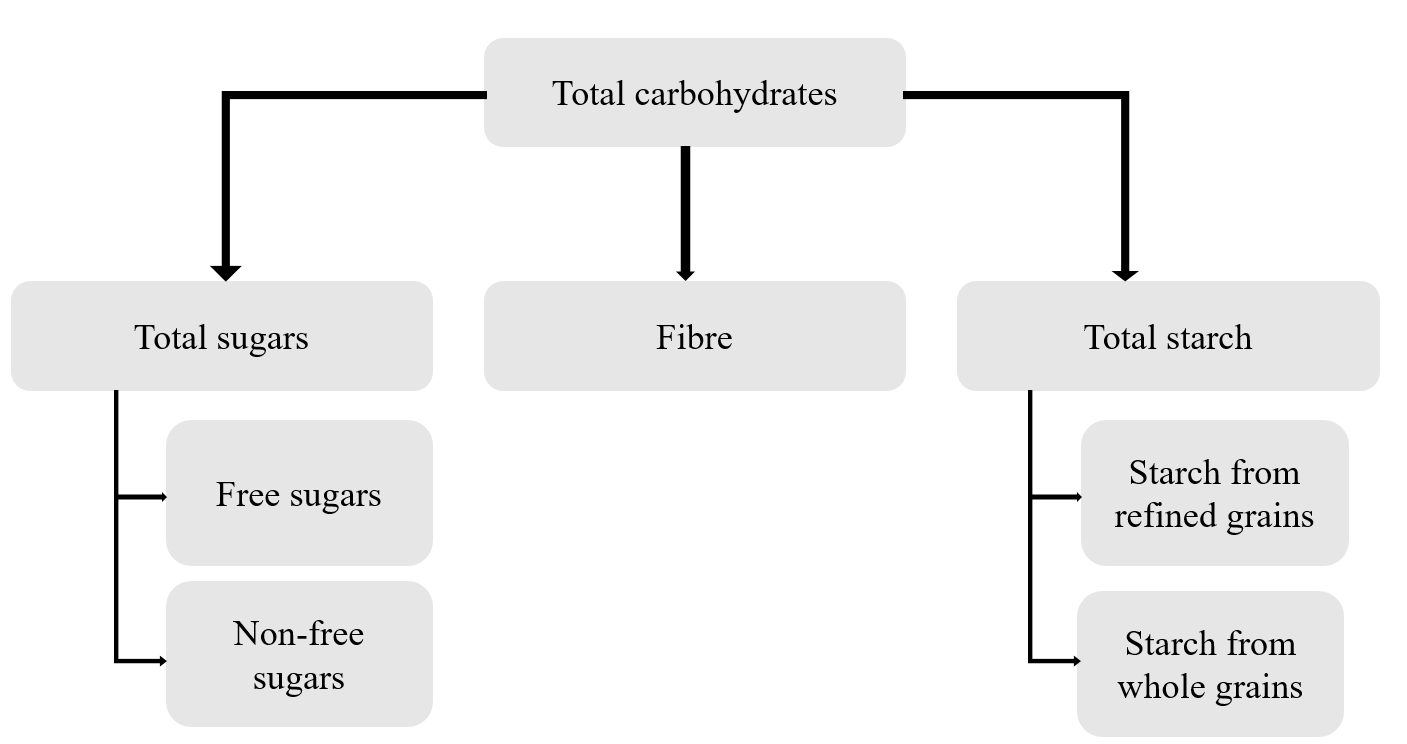


## **Figure S2**. Types and sources of dietary carbohydrates used as exposures for this study


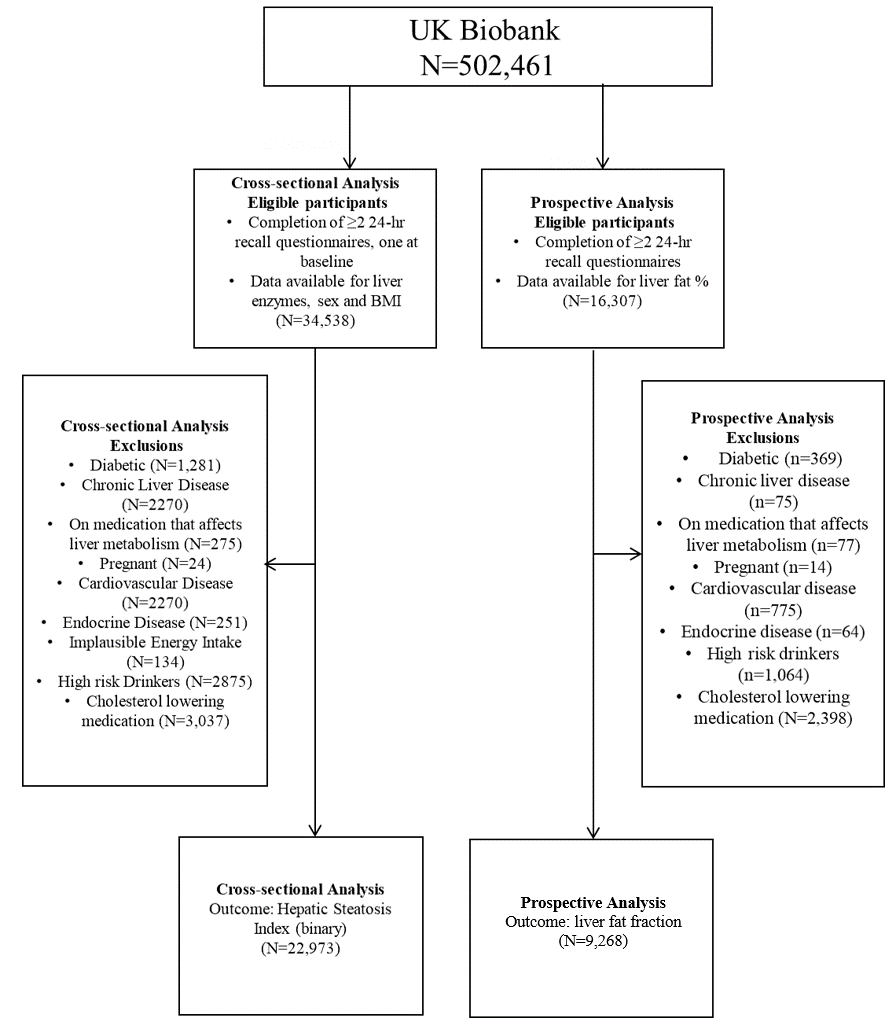


**Figure S3.** Flowchart of UK Biobank showing exclusion of participants included in the cross-sectional and prospective samples
